# An Integrative Pan-Cancer Analysis of the Oncogenic Role of COPB2 in Human Tumors

**DOI:** 10.1155/2021/7405322

**Published:** 2021-10-12

**Authors:** Biao Wu, Yumeng Wu, Xianlin Guo, Yanping Yue, Yuanyuan Li, Xiao He, Yuanbin Chen, Wenjing Zhao, Jibin Liu, Xuming Wu, Aiguo Shen, Suqing Zhang

**Affiliations:** ^1^Department of Hepatobiliary and Pancreatic Surgery, Affiliated Tumor Hospital of Nantong University, China; ^2^Cancer Research Center Nantong, Affiliated Tumor Hospital of Nantong University, China; ^3^Nantong Fourth People's Hospital, China

## Abstract

Several studies have suggested that coatomer protein complex subunit beta 2 (COPB2) may act as an oncogene in various cancer types. However, no systematic pan-cancer analysis has been performed to date. Therefore, the present study analyzed the potential oncogenic role of COPB2 using TCGA (The Cancer Genome Atlas) and GEO (Gene Expression Omnibus) datasets. The majority of the cancer types overexpressed the COPB2 protein, and its expression significantly correlated with tumor prognosis. In certain tumors, such as those found in breast and ovarian tissues, phosphorylated S859 exhibited high expression. It was found that mutations of the COPB2 protein in kidney and endometrial cancers exhibited a significant impact on patient prognosis. It is interesting to note that COPB2 expression correlated with the number of cancer-associated fibroblasts in certain tumors, such as cervical and endocervical cancers and colon adenocarcinomas. In addition, COPB2 was involved in the transport of substances and correlated with chemotherapy sensitivity. This is considered the first pan-tumor study, which provided a relatively comprehensive understanding of the mechanism by which COPB2 promotes cancer growth.

## 1. Introduction

Cancer is the leading cause of death in various countries worldwide, and it is also an essential obstacle to increased life expectancy. The latest statistics have shown that the burden of cancer morbidity and mortality worldwide is constantly increasing [1]. Tumor occurrence is complicated and heterogeneous. With the improvement of the public databases, such as TCGA and GEO, it becomes possible to explore the correlation between genes and clinical prognosis and their related signaling pathways through pan-cancer expression analysis of specific genes [2–4]. In the present study, TCGA and the GEO databases were used to extract information from different tumors, which was used to conduct a pan-cancer analysis.

The coatomer protein complex subunit beta 2 (COPB2) is encoded by a gene on chromosome 3q23. It is one of the non-clathrin-coated vesicular coat subunits that form the coatomer and play a role in membrane transport between the endoplasmic reticulum and Golgi apparatus. COPB2 has a tryptophan-aspartate (WD) repeat sequence associated with signal transduction, cell cycle regulation, and apoptosis [5–8]. Increasing evidence has recently shown that COPB2 plays an important role in tumorigenesis. In several cancer types, COPB2 expression is dysregulated, such as breast [9], bile duct [10], and colon cancers [11]. Numerous experimental studies have confirmed its tumor-promoting function. For example, COPB2 promotes cell proliferation *in vitro* and tumorigenesis *in vivo* by inducing nuclear translocation of YAP1 in lung cancer cells [12].

The present study provides the first pan-cancer analysis for COPB2 using TCGA and the GEO databases. We also included gene expression, survival status, gene mutations, protein phosphorylation, immune infiltration, cellular pathways, and chemotherapy sensitivity analyses in order to explore the underlying molecular mechanisms of COPB2 in the pathogenesis of various cancer types.

## 2. Materials and Methods

### 2.1. Gene Expression Analysis

Initially, the TIMER2 (Tumor Immune Estimation Resource, version 2) web (http://timer.cistrome.org/->CANCER EXPLORATION->Gene_DE) was used to assess COPB2 mRNA expression levels between different tumor tissues and the corresponding paracancerous tissues derived from TCGA database. The GEPIA2 (Gene Expression Profiling Interactive Analysis, version 2) [13] web (http://gepia2.cancer-pku.cn/#analysis->Box Plot) was used to acquire COPB2 mRNA expression level differences between certain tumor tissues from TCGA database that were not paired with normal tissues and the adjacent normal tissues in the GTEx (Genotype-Tissue Expression) database. The following parameter settings were applied: *P* value cut-off = 0.01 and log2FC (fold change) cut‐off = 1. In addition to the mRNA expression levels in tissues, COPB2 mRNA expression levels were assessed in more than one thousand tumor cell lines by the Cancer Cell Line Encyclopedia (CCLE) [14] web (https://www.broadinstitute.org/ccle).

In order to determine the difference in COPB2 protein expression between cancer tissues and the corresponding adjacent tissues of different tumor types, protein expression analysis was performed on the CPTAC (Clinical Proteomic Tumor Analysis Consortium) dataset using the UALCAN [15] (http://ualcan.path.uab.edu/analysis-prot.html->CPTAC analysis). COPB2 (NP_004757.1) expression was assessed with regard to total protein or phosphorylated protein (phosphorylated at T828, S859, and S861 sites) levels between primary tumors and adjacent normal tissues. The available datasets of the six tumors, including breast cancer, ovarian cancer, colon cancer, renal cell cancer, endometrial cancer, and lung adenocarcinomas, were selected. Moreover, the expression levels of the COPB2 protein were assessed in other tumor tissues by HPA [16] (https://www.proteinatlas.org/).

In addition, the “Stage Plot” module of GEPIA2 (http://gepia2.cancer-pku.cn/#survival->Survival Analysis) was used to explore the expression levels of COPB2 in various cancer types at different pathological stages. A cut-off value of 50% was used to divide the groups into the high-expression and low-expression cohorts.

### 2.2. Survival Prognosis Analysis

The “Survival Map” module of GEPIA2 (http://gepia2.cancer-pku.cn/#survival->Survival Map) was used to estimate the OS (overall survival) and DFS (disease-free survival) significance maps of COPB2 in all TCGA tumors. A cut-off value of 50% was used to divide the cohort into high-expression and low-expression groups. Hypothesis testing was performed by the log-rank test.

### 2.3. Genetic Alteration Analysis

Genetic alterations of COPB2 in pan-cancer, including somatic mutations, amplifications, and profound deletions, were evaluated by the cBioPortal for Cancer Genomics [17] (http://www.cbioportal.org). The information of the COPB2 mutation sites can be displayed in the “Mutations” module and in the protein structure schematic or 3D (three-dimensional) structure. Furthermore, OS, DFS, PFS (progression-free survival), and DSS (disease-specific survival) were obtained in TCGA cases with or without COPB2 genetic alterations, and Kaplan-Meier plots were generated with log-rank *P* values.

### 2.4. Immunoinfiltration Analysis

The relationship between COPB2 expression and immune infiltration was examined in all TCGA tumors using the “Immunogene” module of the TIMER2 web server. For this purpose, only cancer-associated fibroblasts (CAFs) and CD8^+^ T cells were used for analysis. Immune infiltration levels were estimated by TIMER, XCELL, QUANTISEQ, MCPCOUNTER, EPIC, CIBERSORT, and CIBERSORT-ABS algorithms. The purity-corrected Spearman rank correlation test obtained *P* values and partial correlation (cor) values. The data were visualized as heat maps and scatter plots.

### 2.5. Functional Enrichment Analysis

Initially, the individual protein name “COPB2” and the organism “Homo sapiens” were searched using the STRING website [18] (https://string-db.org/). Subsequently, the following parameters were set: minimum required interaction score (“low confidence (0.150)”), meaning of network edges (“evidence”), max number of interactors to show (“no more than 50 interactors”), and active interaction sources (“experiments”). Finally, 50 proteins were obtained that could bind to COPB2.

GEPIA2's “Similar Gene Detection” module was used to obtain the top 100 target genes associated with COPB2 in TCGA dataset. In addition, Pearson correlation analysis of selected genes was performed using GEPIA2's “correlation analysis” module. The *P* values and correlation coefficients (*R*) are provided. Furthermore, heat maps were provided for the selected genes using the “Gene_Corr” module of TIMER2, which included the biased correlation (cor) and *P* values calculated from Spearman's rank correlation test or a purity adjustment.

The genes interacting with COPB2 by Jvenn [19] were cross-tabulated by an interactive Venn diagram viewer. In addition, the two datasets were combined for Gene Ontology (GO) analysis and KEGG (Kyoto Encyclopedia of Genes and Genomes) pathway analysis with OmicShare tools (http://www.omicshare.com/tools). GO and KEGG entries with a false discovery rate (FDR) and corrected *P* values less than 0.05 were considered significantly enriched. The top 20 GO and KEGG pathway terms were visualized as bubble plots.

## 3. Results

### 3.1. Analysis of COPB2 Expression in Tumor and Nontumor Tissues

**Figure 1:**
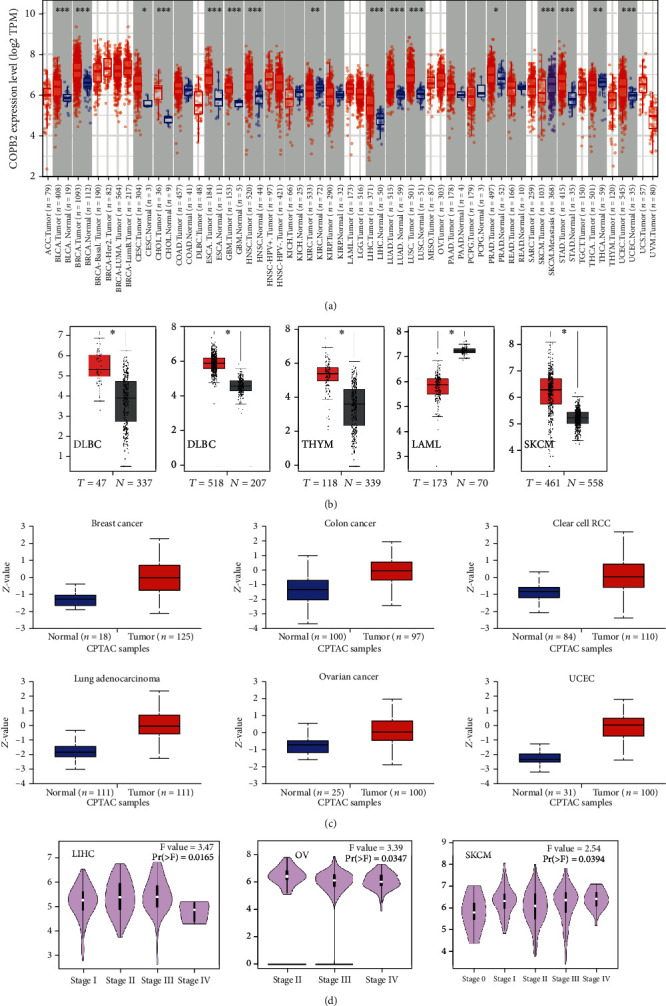
Expression of the COPB2 gene in different cancer types and pathological tumor stages. (a) Analysis of COPB2 mRNA expression in different tumors by TIMER2. ^∗^*P* < 0.05; ^∗∗^*P* < 0.01; ^∗∗∗^*P* < 0.001. (b) Differential mRNA expression of COPB2 in DLBC, LGG, THYM, LAML, and SKCM versus the corresponding nontumor tissues in TCGA dataset. The box chart data are provided. ^∗^*P* < 0.05. (c) The protein expression levels of COPB2 were analyzed in tumor and paracancerous tissues of several cancers (breast cancer, colon cancer, clear cell RCC, LUAD, ovarian cancer, and UCEC) by the CPTAC dataset. (d) In addition, the differences in COPB2 expression levels were analyzed in the pathological stages of different tumors (LIHC, OV, and SKCM). Log2 (TPM + 1) was used for logarithmic analysis.

Initially, the expression pattern of COPB2 was analyzed in different cancer types derived from TCGA database by the TIMER method. In Figure 1(a), COPB2 expression was higher in BLCA (bladder urothelial carcinoma) (*P* < 0.001), BRCA (breast invasive carcinoma), CESC (cervical squamous cell carcinoma and endocervical adenocarcinoma) (*P* < 0.05), CHOL (cholangiocarcinoma), ESCA (esophageal carcinoma), GBM (glioblastoma multiforme), HNSC (head and neck squamous cell carcinoma), KIRC (kidney renal clear cell carcinoma) (*P* < 0.01), LIHC (liver hepatocellular carcinoma), LUAD (lung adenocarcinoma), LUSC (lung squamous cell carcinoma), PRAD (prostate adenocarcinoma), STAD (stomach adenocarcinoma), THCA (thyroid carcinoma), and UCEC (uterine corpus endometrial carcinoma) than in the corresponding nontumor tissues.

Subsequently, the differences in COPB2 expression were assessed between normal tissues and DLBC (lymphoid neoplasm diffuse large B cell lymphoma) (*P* < 0.05), LGG (brain lower grade glioma), LAML (acute myeloid leukemia), SKCM (skin cutaneous melanoma), and THYM (thymoma) tissues (Figure 1(b)).

Surprisingly, in LAML (*P* < 0.05), COPB2 expression was low and differentially expressed in cancerous tissues. Moreover, for other tumors, such as ACC (adrenocortical carcinoma), HNSC (head and neck squamous cell carcinoma), OV (ovarian serous cystadenocarcinoma), SARC (sarcoma), TGCT (testicular germ cell tumors), and UCS (uterine carcinosarcoma), nonsignificant differences were obtained (Figure S1). The mRNA expression levels of specific cancer cell lines were examined using the Cancer Cell Line Encyclopedia (CCLE), and the data indicated that COPB2 expression levels were highly expressed in almost all tumor cell lines, notably neurological tumors (Figure S2).

At the protein level, the results from the CPTAC dataset indicated that the expression levels of total COPB2 protein were higher in tissues of breast cancer, ovarian cancer, colon cancer, clear cell RCC, UCEC, and LUAD than in nontumor tissues (Figure 1(c), *P* < 0.001). In addition, data from the Human Protein Atlas (HPA) indicated that COPB2 was stably expressed in almost all patients with thyroid, lung, colon, head and neck, and breast cancers (Figure S3). In conclusion, COPB2 indicated a pattern of upregulation in the majority of the cancer types, suggesting that it may be a potential tumor promoter.

The “pathological staging map” module of GEPIA2 was applied to explore whether COPB2 expression may differ in different pathological stages of tumors. The outcomes indicated that COPB2 expression levels were significantly associated with the clinical stage of the following cancer types: LIHC (*P* = 0.0165), OV (*P* = 0.0347), and SKCM (*P* = 0.0394) (Figure 1(d)). The expression levels of COPB2 continued to increase following the increase in the LIHC and SKCM tumor grade, further emphasizing its potential tumor-promoting function in these cancer types. However, it is noteworthy that the expression levels of COPB2 were decreased with the increasing tumor stage in OV patients.

### 3.2. Correlation between COPB2 Expression and Prognostic Significance in Various Cancer Types

**Figure 2:**
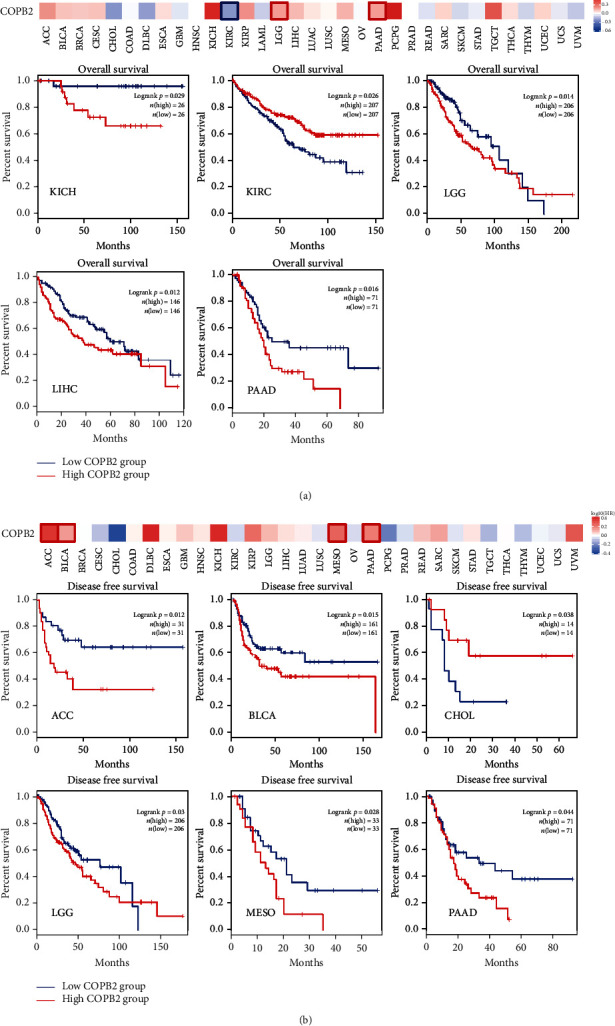
The relationship between the expression of COPB2 and the prognosis of cancer types. The overall survival (a) and disease-free survival (b) of diverse cancer types were assessed based on COPB2 expression in TCGA database. Moreover, the data indicated several Kaplan-Meier curves that produced conclusive results.

Considering the significant dysregulation of COPB2 expression in certain cancer types and its correlation with the tumor stage, it was speculated that this protein may be used as a cancer prognostic indicator. The tumor samples were divided into high- and low-expression groups based on the expression levels of COPB2 and the association between COPB2 expression and prognostic significance with different cancer types derived from TCGA database. As shown in Figure 2(a), in TCGA project, high expression of COPB2 was associated with poor OS prognosis in KICH (*P* = 0.029), LGG (*P* = 0.014), LIHC (*P* = 0.012), and PAAD (*P* = 0.016). The data from DFS analysis (Figure 2(b)) indicated that high expression of COPB2 was interrelated with poor prognosis of TCGA cases with ACC (*P* = 0.012), BLCA (*P* = 0.015), LGG (*P* = 0.03), MESO (*P* = 0.028), and PAAD (*P* = 0.044) tumors. In contrast to these findings, low COPB2 expression was associated with poor prognosis of OS for KIRC (*P* = 0.0026) and poor prognosis of DFS for CHOL (*P* = 0.038).

### 3.3. The Genetic Alteration of COPB2 in Pan-Cancer Datasets

**Figure 3:**
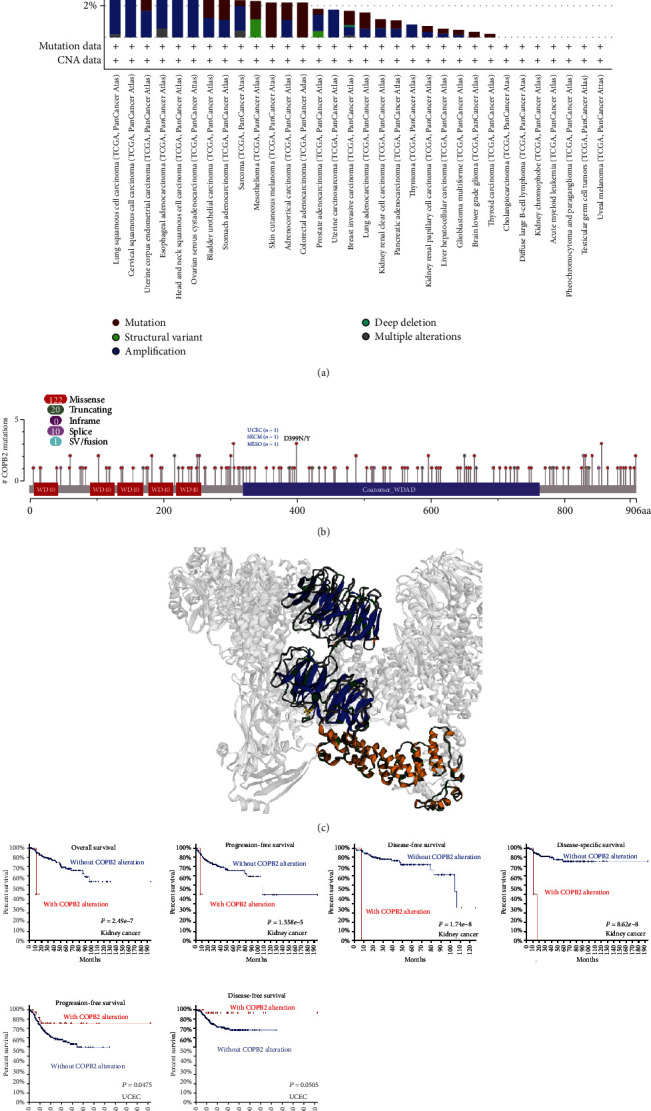
Mutation characteristics of COPB2 in various cancer types. The mutation types of different tumors (a) and the frequency of mutation sites (b) were displayed using the cBioPortal tool. (c) In addition, the analysis indicated the most frequent mutation sites (D399N/Y) in the 3D structure pattern of the COPB2 coatomer region. Possible relationships between mutational status and overall, disease-specific, disease-free, and progression-free survival of UCEC and KIRC (d) were analyzed.

Subsequently, the genetic alterations of COPB2 were investigated among different cancer samples from TCGA database. A relatively low overall mutation rate of COPB2 was observed in all cancer types (less than 10%). As shown in Figure 3(a), the highest frequency of COPB2 alterations (>5%) was observed in UCEC patients with a “mutation” as the primary feature, followed by BLCA. The “amplification” type of CNA was the main type of LUSC, and the frequency of change was approximately 8%. The cBioPortalOncoprint indicated that amplification was the main type of mutation noted in COPB2 (Figure S4).

Although a mutational hotspot for COPB2 was not identified in the pan-cancer dataset, the highest alteration frequency in the COA region was identified in the 3D model of COPB2 (Figures 3(b) and 3(c)). Furthermore, the potential association between COPB2 gene alterations and the prognosis of different cancer patients was explored. The image in Figure 3(d) indicated that COPB2-altered UCEC patients indicated an improved prognosis in PFS (progression-free survival) (*P* = 0.0475), but not DFS (*P* = 0.0505), compared with those noted in COPB2-unaltered cases. Conversely, patients with COPB2 alterations in kidney cancer exhibited worse prognosis in terms of OS (*P* = 2.49*e* − 7), DFS (*P* = 1.74*e* − 8), PFS (*P* = 1.558*e* − 5), and DSS (*P* = 8.62*e* − 8). The aforementioned data suggested that the presence of mutations in the COPB2 gene differed with regard to the prognosis of patients with various cancer types.

### 3.4. Analysis of Protein Phosphorylation Levels of COPB2

**Figure 4:**
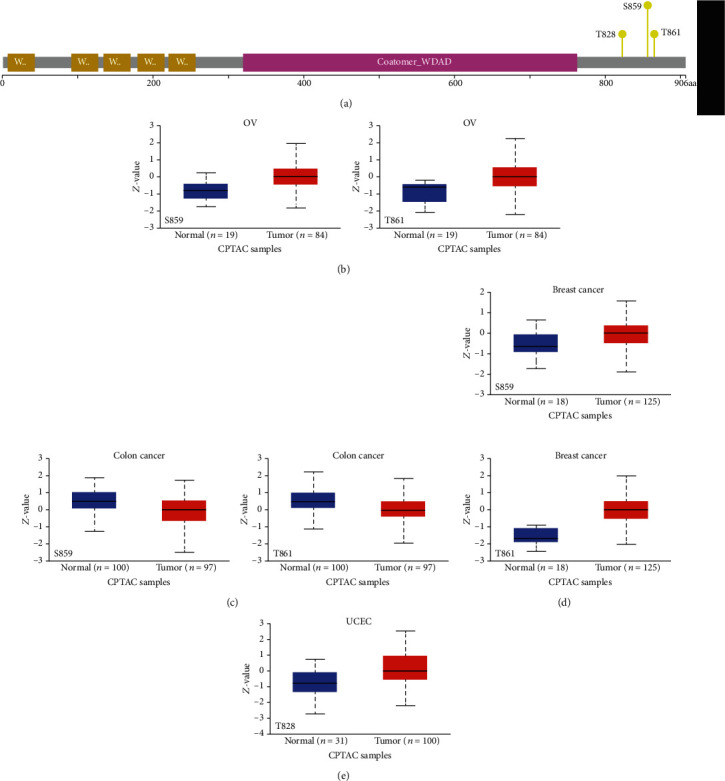
Analysis of COPB2 protein phosphorylation in various cancer types. (a) Three phosphorylated protein sites (T828, S859, and T861) of COPB2 were confirmed using the cBioPortal tool. (b–e) The expression levels of the COPB2 phosphoprotein were examined between tumor tissues and the corresponding normal tissues using UALCAN software. Furthermore, several box plots were produced demonstrating significant results regarding the association of COPB2 expression and the incidence of several cancer types (ovarian cancer, colon cancer, breast cancer, and UCEC).

The differences in the levels of COPB2 phosphorylation between tumor tissues and the corresponding normal tissues were examined. Four types of cancers (breast cancer, colon cancer, OC, and UCEC) were analyzed using the CPTAC dataset.  Figure 4(a) indicates three COPB2 phosphorylation sites. The S859 and T861 sites indicated higher phosphorylation levels in OV and breast cancer tissues compared with those noted in normal tissues, while the opposite results were obtained in colon cancer samples (Figures 4(b)–4(d)). In addition, the T828 locus was also expressed at significantly increased levels in UCEC (Figure 4(e)).

### 3.5. Analysis of Protein Methylation Expression of COPB2

Furthermore, the differences in COPB2 methylation levels between tumor tissues and corresponding normal tissues were assessed. Supplemental Figure S5 demonstrates that the methylation levels of COPB2 were reduced in tumor tissues compared with those noted in normal tissues (BLCA, HNSC, KRIP, LIHC, PRAD, TGCT, and UCEC) (*P* < 0.05). This finding warrants further investigations in order to explore the potential role of COPB2 methylation in tumorigenesis.

### 3.6. Relationship between COPB2 Expression and Immune Infiltration in Cancer

**Figure 5:**
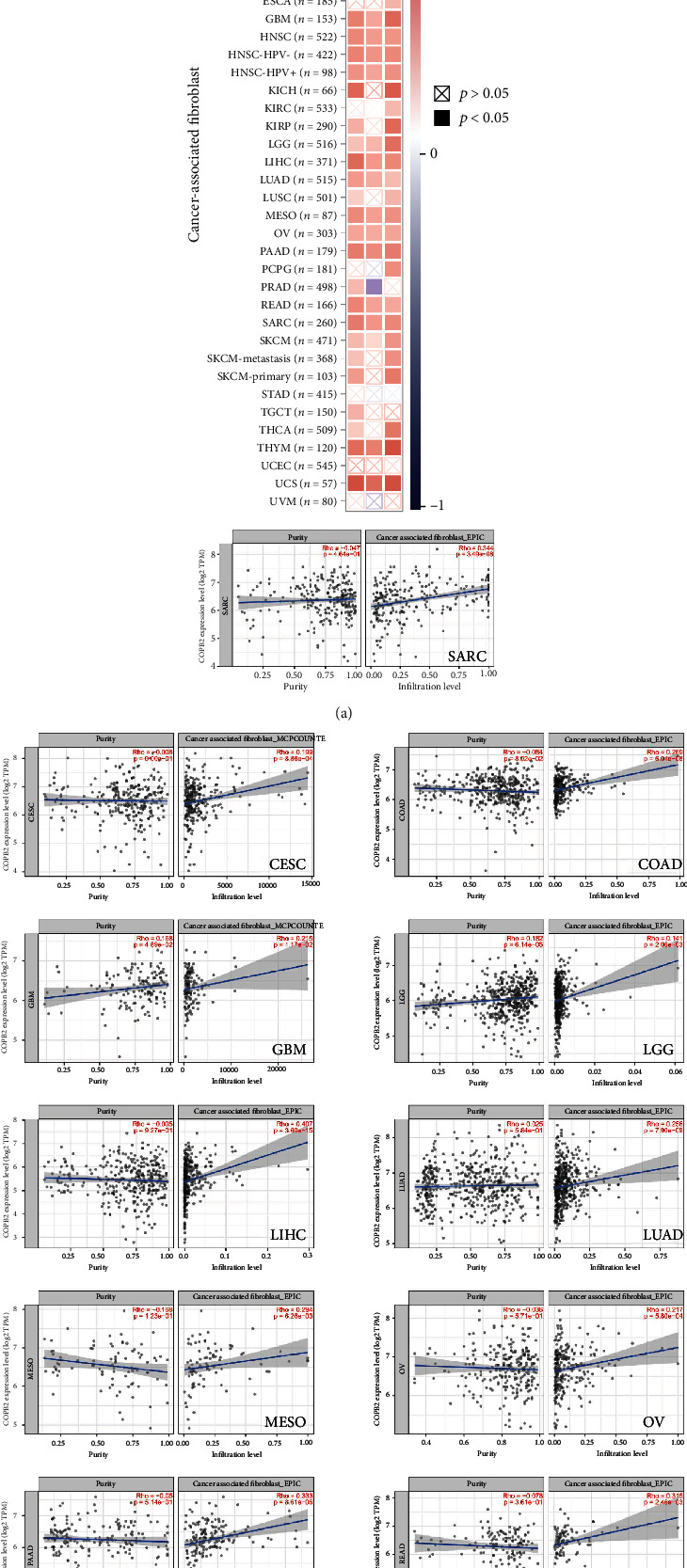
The relationship between COPB2 expression and immune infiltration of cancer-associated fibroblasts (CAFs). (a) Three algorithms (EPIC, MCPCOUNTER, and TIDE) were used to investigate the possible relationship between COPB2 expression and infiltration of cancer-associated fibroblasts in various cancer types. (b) Moreover, the results yielded appropriate conclusions.

As an essential component of the tumor microenvironment, tumor-infiltrating immune cells are closely associated with tumorigenesis, progression, or metastasis. Tumor-associated fibroblasts in the tumor microenvironment stroma have been reported to regulate the functions of a variety of tumor-infiltrating immune cells. Therefore, it is necessary to discuss the relationship between COPB2 expression and pro/antitumor immune components. In the present study, a total of seven algorithms (EPIC, MCPCOUNTER, CIBERSORT, CIBERSORT-ABS, QUANTISEQ, XCELL, and TIDE) was used to explore the underlying association between different levels of immune cell infiltration and COPB2 expression in different cancer types derived from TCGA database. Six algorithms (EPIC, MCPCOUNTER, CIBERSORT, CIBERSORT-ABS, QUANTISEQ, and XCELL) were used to quantify the density of CD8^+^T cells in each cancer type. Subsequently, correlation analysis was performed with the expression levels of COPB2. An overall negative correlation between CD8^+^T cells and COPB2 expression was noted in pan-cancerous tissues, with the exception for DLBC, LGG, and UVM (Figure S6). Cancer-associated fibroblasts (CAFs) are often considered to exert protumorigenic properties. Based on these three algorithms (EPIC, MCPCOUNTER, and TIDE), the analysis indicated that COPB2 expression and CAF abundance were positively correlated in the majority of the cancer types (Figure 5(a)). As shown in Figure 5(b), COPB2 expression was positively associated with the infiltration of cancer-associated fibroblasts in CESC, COAD, GBM, HNSC, LGG, LIHC, LUAD, MESO, OV, PAAD, READ, SARC, THYM, and UCS cancer types derived from TCGA database. The aforementioned tumor scatter plot data were obtained using one algorithm (Figure 5(b)). For example, based on the MCPCOUNTER algorithm, the COPB2 expression of CESCs was positively associated with the infiltration level of cancer-associated fibroblasts (cor = 0.199, *P* = 9.86*e* − 4).

### 3.7. Enrichment Analysis of COPB2 and Related Proteins

**Figure 6:**
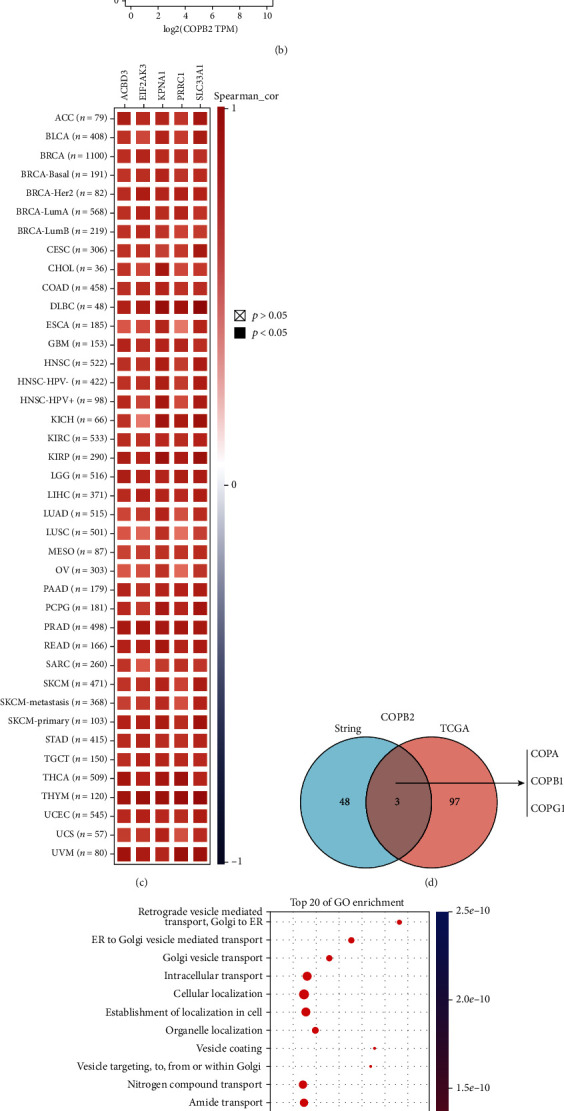
Enrichment analysis of the COPB2 gene. (a) A total of 50 proteins that bind to COPB2 were identified using the STRING tool. (b) In addition, 100 genes associated with COPB2 were acquired from TCGA database, and the data demonstrated the relevance of five genes (ACBD3, EIF2AK3, KPNA1, PRRCC1, and SLC33A1) interacting with COPB2. (c) The association of 5 genes with the incidence of various cancer types was examined. (d) The cross-tabulation of the genes was obtained from these two datasets, and GO analysis (e) and KEGG pathway analysis (f) were performed.

To further explore the potential molecular mechanisms of COPB2 in tumorigenesis, relevant genes targeting COPB2 expression were selected and a string of enrichment analysis was performed. Using the STRING tool, 50 binding proteins were identified for the COPB2 gene, and Figure 6(a) indicates the network of the interactions of these genes. Subsequently, the GEPIA2 tool was used to assess all TCGA tumor expression data in order to obtain the top 100 genes associated with COPB2 expression. As shown in Figure 6(b), COPB2 expression levels were associated with ACBD3 (acyl-coenzyme A-binding domain containing 3 protein) (*R* = 0.7), EIF2AK3 (eukaryotic translation initiation factor 2-alpha kinase 3) (*R* = 0.67), KPNA1 (karyopherin alpha 1) (*R* = 0.69), PRRC1 (proline-rich coiled-coil 1) (*R* = 0.67), and SLC33A1 (acetyl-CoA transporter) (*R* = 0.7) (all *P* < 0.001). In most detailed cancer types, the corresponding heat map further demonstrated a positive relationship of COPB2 with the aforementioned five genes (Figure 6(c)). The aforementioned two datasets exhibited 3 members in common, namely, COPA (coatomer subunit alpha), COPB1 (coatomer protein complex, subunit beta 1), and COPG1 (coatomer protein complex subunit *γ*1) (Figure 6(d)).

These two datasets were joined for KEGG and GO enrichment analyses. GO pathway analysis indicated that COPB2 was mainly associated with substance transport, such as “retrograde vesicle-mediated transport, Golgi to ER,” “ER to Golgi vesicle-mediated transport,” “Golgi vesicle transport,” and “intracellular transport” (Figure 6(e)). The KEGG data in Figure 6(f) suggested that the effects of COPB2 on tumorigenesis may be associated with “pathogenic Escherichia coli infection,” “gap junction,” and “protein processing in the endoplasmic reticulum.”

The GSCALite webserver was used to analyze the association between COPB2 and drug sensitivity. As shown in Supplemental Figure S7, high COPB2 expression levels were associated with high sensitivity to vorinostat, NPK76-II-72-1, GSK1070916, TPCA-1, and navitoclax and conversely with low sensitivity to TGX221 and docetaxel.

## 4. Discussion

In the 21st century, cancer is considered a main factor responsible for patient death globally. The rapid increase in cancer incidence and mortality has led to the development of effective tumor prevention and intervention strategies. A limited number of studies in recent years have reported the relationship between COPB2 and specific common diseases [20], notably cancer [21]. Whether COPB2 can play an important role in different cancer types via specific mechanisms remains to be studied. However, a potential interaction has not been previously reported between COPB2 expression and pan-cancer. Therefore, the current study examined COPB2 gene expression in 33 different cancer types based on TCGA, GEO, and CPTAC database data combined with molecular features of gene expression, gene mutation, or protein phosphorylation in a comprehensive manner.

Previous studies have shown that COPB2 is abnormally expressed in the vast majority of tumors and that it is related to the poor prognosis of patients. In a recent liver cancer study, the database of specific patient data was used and the data demonstrated that patients with high COPB2 expression exhibited a poorer prognosis [22]. In the present study, the GEPIA2 tool was used to assess the association of the OS and DFS in patients with high COPB2 expression in pan-cancer tissues. Poor prognosis was associated with low survival and high COPB2 expression. However, specific exceptions were noted, such as the ability of high COPB2 expression to predict improved OS in KIRC.

The present study demonstrated that COPB2 could affect multiple mechanisms that participated in tumor progression. The first is the mutation-driven mechanism. The analysis indicated that the frequency of missense mutations was highest in UCEC, whereas the frequency of amplification mutations was the highest in LUSC. In addition, COPB2 mutations affected patient prognosis; in UCEC, COPB2 mutations prolonged PFS, whereas in KIRC, COPB2 mutations significantly decreased OS, PFS, DFS, and DSS. This result led to the conclusion that low COPB2 expression was associated with poor prognosis of KIRC patients, which may be caused by gene mutations.

The underlying molecular mechanism of COPB2 was assessed in breast and colon cancers, OV, and UCEC with regard to total and phosphorylated protein levels. The results indicated that the phosphorylation sites of COPB2 exhibited an increasing trend in breast cancer, OV, and UCEC, whereas a decreasing trend was noted in colon cancer. However, the correlation between COPB2 phosphorylation modification and tumorigenesis has not been previously reported, and additional experiments are urgently required to explore this hypothesis. In addition to the level of COPB2 phosphorylation, the correlation between COPB2 methylation and tumor incidence was examined. COPB2 methylation levels were significantly reduced in BLCA, HNSC, KRIP, LIHC, PRAD, TGCT, and UCEC. It is well known that the immune microenvironment plays a significant role in both cancer progression and elimination [23]. The results of the present study further suggest for the first time the link between COPB2 expression and the immune infiltration of cancer-associated fibroblasts in certain tumors.

Finally, the current analysis aided the identification of the underlying functional role of COPB2 in tumors. By using GO and KEGG analysis, the data indicated that COPB2 mainly played a role in protein transport. This was not surprising since COPB2 acted as a subunit of the COPI complex participating in membrane and protein reverse transport between the endoplasmic reticulum and the Golgi apparatus [24, 25]. This function of COPI plays a necessary role in cancer development. For example, the epidermal growth factor receptor (EGFR) is localized in the nucleus, and nuclear translocation of EGFR is dependent on COPI transport [26], which is involved in DNA repair, transcriptional regulation, cell proliferation, and other processes. In addition, COPI is also involved in the drug delivery pathway to the cell interior, which is one of the possible reasons for the association of COPB2 with drug sensitivity [27]. However, the correlation of COPB2 with drug sensitivity has not been previously reported; therefore, it is of great interest to determine the relationship between COPB2 and drug sensitivity. The relationship between COPB2 and certain drugs was also explored at a preliminary level. This can be used as a future reference for subsequent experiments.

The present study has some limitations. The majority of the conclusions was drawn by bioinformatic analysis. Therefore, the current study lacked a rigorous mechanistic explanation supported by experimental data, and further studies are required to validate these results and investigate the biological functions of COPB2 in various tumors. For this purpose, a large sample size and independent validation of these findings would be required in order to produce reliable and generalizable conclusions.

## 5. Conclusions

In conclusion, the first pan-cancer analysis of COPB2 indicated that COPB2 expression was significantly correlated with prognosis of cancer patients, genetic alteration, immune cell infiltration, and drug sensitivity in various tumors. COPB2 acted as a tumor promoter in the majority of the tumors investigated and can be a potential marker of cancer prognosis. This contributes to our understanding of the role of COPB2 in tumorigenesis. In future research, we should explore the mechanisms by which COPB2 promotes tumorigenesis, such as gene mutations, gene modifications, and related signaling pathways. In terms of cancer treatment, we should focus on exploring the role of COPB2 in immunotherapy and targeted therapy.

## Data Availability

The datasets analyzed during the current study are available in TCGA (https://portal.gdc.cancer.gov/) and GEO repository (https://www.ncbi.nlm.nih.gov/geo/).

## Supplementary Materials

Supplementary Materials Figure S1. COPB2 mRNA expression in tumor tissues (ACC, HNSC, OV, SARC, TGCT, and UCS) and the corresponding nontumor tissues. Figure S2. Protein expression of COPB2 in tumor patients in the HPA (Human Protein Atlas) dataset. Figure S3. mRNA expression levels of COPB2 in different types of tumor cells from the Cancer Cell Line Encyclopedia (CCLE) database. Figure S4. cBioPortalOncoprint demonstrating the mutation spectrum of COPB2 across TCGA pan-cancer studies. Each vertical bar represents a patient. Figure S5. Expression of COPB2 phosphoprotein between tumor tissues and the corresponding normal tissues through UALCAN. Figure S6. Associations between COPB2 expression levels and immune infiltration of CD8^+^T cells across different cancer types. Figure S7. Relationship between COPB2 expression and drug sensitivity from the GSCALite web.

## References

[1] Sung H., Ferlay J., Siegel R. L. (2021). Global cancer statistics 2020: GLOBOCAN estimates of incidence and mortality worldwide for 36 cancers in 185 Countries. *CA: a Cancer Journal for Clinicians*.

[2] Blum A., Wang P., Zenklusen J. C. (2018). SnapShot: TCGA-analyzed tumors. *Cell*.

[3] Lee H. J., Palm J., Grimes S. M., Ji H. P. (2015). The Cancer Genome Atlas Clinical Explorer: a web and mobile interface for identifying clinical-genomic driver associations. *Genome Medicine*.

[4] Zhu Y., Davis S., Stephens R., Meltzer P. S., Chen Y. (2008). GEOmetadb: powerful alternative search engine for the Gene Expression Omnibus. *Bioinformatics*.

[5] Orci L., Palmer D. J., Amherdt M., Rothman J. E. (1993). Coated vesicle assembly in the Golgi requires only coatomer and ARF proteins from the cytosol. *Nature*.

[6] Presley J. F., Ward T. H., Pfeifer A. C., Siggia E. D., Phair R. D., Lippincott-Schwartz J. (2002). Dissection of COPI and Arf1 dynamics _in vivo_ and role in Golgi membrane transport. *Nature*.

[7] Beck R., Ravet M., Wieland F. T., Cassel D. (2009). The COPI system: molecular mechanisms and function. *FEBS Letters*.

[8] Lee M. C. S., Miller E. A., Goldberg J., Orci L., Schekman R. (2004). Bi-directional protein transport between the ER and Golgi. *Annual Review of Cell and Developmental Biology*.

[9] Bhandari A., Zheng C., Sindan N. (2019). COPB2 is up-regulated in breast cancer and plays a vital role in the metastasis via N-cadherin and vimentin. *Journal of Cellular and Molecular Medicine*.

[10] Wang Y., Chai Z., Wang M., Jin Y., Yang A., Li M. (2018). COPB2 suppresses cell proliferation and induces cell cycle arrest in human colon cancer by regulating cell cycle-related proteins. *Experimental and Therapeutic Medicine*.

[11] Li Z. S., Liu C. H., Liu Z., Zhu C.-L., Huang Q. (2018). Downregulation of COPB2 by RNAi inhibits growth of human cholangiocellular carcinoma cells. *European Review for Medical and Pharmacological Sciences*.

[12] Pu X., Wang J., Li W. (2018). COPB2 promotes cell proliferation and tumorigenesis through up-regulating YAP1 expression in lung adenocarcinoma cells. *Biomedicine & Pharmacotherapy*.

[13] Tang Z., Kang B., Li C., Chen T., Zhang Z. (2019). GEPIA2: an enhanced web server for large-scale expression profiling and interactive analysis. *Nucleic Acids Research*.

[14] Barretina J., Caponigro G., Stransky N. (2012). The Cancer Cell Line Encyclopedia enables predictive modelling of anticancer drug sensitivity. *Nature*.

[15] Chen F., Chandrashekar D. S., Varambally S., Creighton C. J. (2019). Pan-cancer molecular subtypes revealed by mass-spectrometry-based proteomic characterization of more than 500 human cancers. *Nature Communications*.

[16] Kampf C., Bergman J., Oksvold P. (2012). A tool to facilitate clinical biomarker studies--a tissue dictionary based on the Human Protein Atlas. *BMC Medicine*.

[17] Gao J., Aksoy B. A., Dogrusoz U. (2013). Integrative analysis of complex cancer genomics and clinical profiles using the cBioPortal. *Science Signaling*.

[18] Szklarczyk D., Gable A. L., Lyon D. (2019). STRING v11: protein-protein association networks with increased coverage, supporting functional discovery in genome-wide experimental datasets. *Nucleic Acids Research*.

[19] Bardou P., Mariette J., Escudié F., Djemiel C., Klopp C. (2014). Jvenn: an interactive Venn diagram viewer. *BMC Bioinformatics*.

[20] DiStasio A., Driver A., Sund K. (2017). Copb2 is essential for embryogenesis and hypomorphic mutations cause human microcephaly. *Human Molecular Genetics*.

[21] Wang Y., Xie G., Li M., Du J., Wang M. (2020). COPB2 gene silencing inhibits colorectal cancer cell proliferation and induces apoptosis via the JNK/c-Jun signaling pathway. *PLoS One*.

[22] Zhang J., Wang X., Li G. (2021). COPB2: a novel prognostic biomarker that affects progression of HCC. *BioMed Research International*.

[23] Zhu Y., Zhang Z., Jiang Z., Liu Y., Zhou J. (2020). CD38 predicts favorable prognosis by enhancing immune infiltration and antitumor immunity in the epithelial ovarian cancer microenvironment. *Frontiers in Genetics*.

[24] Béthune J., Wieland F., Moelleken J. (2006). COPI-mediated transport. *Journal of Membrane Biology*.

[25] Dodonova S. O., Diestelkoetter-Bachert P., von Appen A. (2015). A structure of the COPI coat and the role of coat proteins in membrane vesicle assembly. *Science*.

[26] Wang Y.-N., Wang H., Yamaguchi H., Lee H.-J., Lee H.-H., Hung M.-C. (2010). COPI-mediated retrograde trafficking from the Golgi to the ER regulates EGFR nuclear transport. *Biochemical and Biophysical Research Communications*.

[27] Tarragó-Trani M. T., Storrie B. (2007). Alternate routes for drug delivery to the cell interior: pathways to the Golgi apparatus and endoplasmic reticulum. *Advanced Drug Delivery Reviews*.

