# Supplementary material for: An Integrative Pan-Cancer Analysis of the Oncogenic Role of COPB2 in Human Tumors
Source: Biomed Res Int. 2021 Oct 12;2021:7405322. doi: 10.1155/2021/7405322 (PMC8526247; doi:10.1155/2021/7405322)

PROTEIN EXPRESSION SUMMARY[i]

Organ | Expression | Alphabetical

HPA036867

Malignant cells exhibited moderate immunoreactivity. A few cases of malignant carcinoids, endometrial, colorectal and prostate cancers were strongly stained. Several cases of renal and liver cancers were negative.

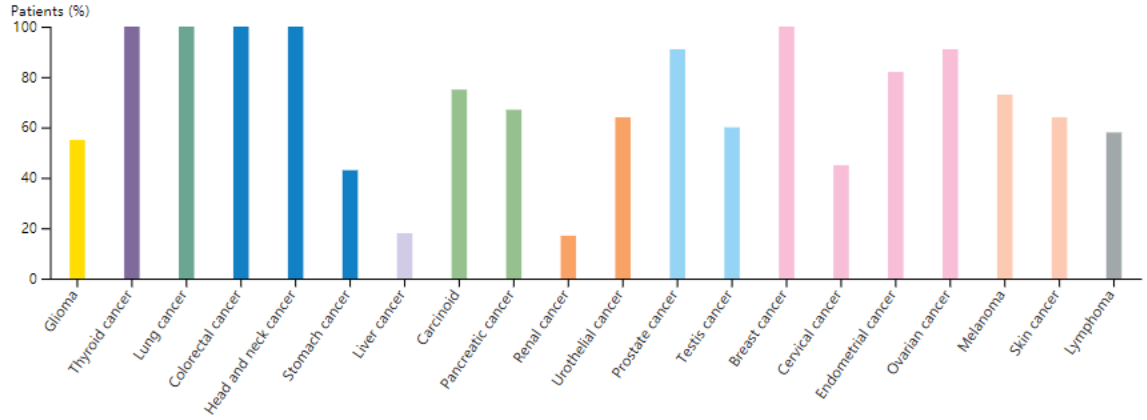

Supplement: Supplementary Materials — Figure S1. COPB2 mRNA expression in tumor tissues (ACC, HNSC, OV, SARC, TGCT, and UCS) and the corresponding nontumor tissues. Figure S2. Protein expression of COPB2 in tumor patients in the HPA (Human Protein Atlas) dataset. Figure S3. mRNA expression levels of COPB2 in different types of tumor cells from the Cancer Cell Line Encyclopedia (CCLE) database. Figure S4. cBioPortalOncoprint demonstrating the mutation spectrum of COPB2 across TCGA pan-cancer studies. Each vertical bar represents a patient. Figure S5. Expression of COPB2 phosphoprotein between tumor tissues and the corresponding normal tissues through UALCAN. Figure S6. Associations between COPB2 expression levels and immune infiltration of CD8+T cells across different cancer types. Figure S7. Relationship between COPB2 expression and drug sensitivity from the GSCALite web. [file 7405322.f1.zip › 7405322.f2.pdf]
